# LYN and CYBB are pivotal immune and inflammatory genes as diagnostic biomarkers in recurrent spontaneous abortion

**DOI:** 10.3389/fimmu.2025.1568536

**Published:** 2025-07-07

**Authors:** Zhuna Wu, Qiuya Lin, Zhimei Zhou, Yajing Xie, Li Huang, Liying Sheng, Qirong Shi, Yumin Ke

**Affiliations:** Department of Gynecology and Obstetrics, The Second Affiliated Hospital of Fujian Medical University, Quanzhou, Fujian, China

**Keywords:** recurrent spontaneous abortion, cybb, lyn, inflammation, immunity

## Abstract

Recurrent spontaneous abortion (RSA) seriously affects women’s reproductive health, and its pathogenesis is complex and varied. The aim of this study is to identify key molecular markers closely associated with RSA to rapidly and effectively predict the RSA, and to provide simple and practical indicators for clinical diagnosis and treatment.

**Method:**

We obtained mRNA expression profiles from the GSE26787 and GSE165004 datasets of the Gene Expression Omnibus (GEO) database, immune-related genes (IRGs) from the ImmPort database (https://www.immport.org), and genes related to inflammatory response from the Molecular Signatures database. Different Inflammation- and immunity-related genes (DIIRGs) were subjected to Gene Ontology (GO) and Kyoto Encyclopedia of Genes and Genomes (KEGG) analysis. Protein-protein interaction (PPI) networks were utilized to explore the connections between various DIIRGs. The candidate DIIRGs were analyzed by the least absolute shrinkage and selection operator (LASSO) and the multiple support vector machine recursive feature elimination (mSVM-RFE). The diagnostic ability of the candidate genes was verified using receiver operating characteristic (ROC) curves. The performance of the predictive model was evaluated using a Nomo plot. We further confirmed the expression levels and diagnostic value of key genes by performing immunohistochemistry (IHC) in clinical tissue samples. The compositional patterns of the infiltration of 22 immune cell types in RSA were analyzed via the CIBERSORT algorithm.

**Result:**

We identified 403 differentially expressed genes (DEGs) and 7 DIIRGs between RSA endometrium and Non-RSA endometrium. GO analysis showed that DIIRGs were significantly enriched in positive regulation of cell-cell adhesion, inflammatory response to antigenic stimulus, and protein tyrosine kinase activity. KEGG enrichment analyses were performed mainly on Epithelial cell signaling in Helicobacter pylori infection, NOD-like receptor signaling pathway, and Ras signaling pathway. A predictive and diagnostic model composed of three genes (CYBB, LYN, and MET). The CYBB, LYN, and MET genes were identified as diagnostic biomarkers of RSA (AUC = 0.747, AUC = 0.751, AUC=0.703), and reduced levels of CYBB and LYN expression were found to correlate with RSA in clinical samples. In addition, immune microenvironment analysis showed that CYBB and MET were positively correlated with naïve B cells and negatively correlated with CD8 T cells, LYN and MET were positively correlated with M2 macrophages and negatively correlated with eosinophils, respectively (P < 0.05).

**Conclusion:**

Inflammation-immunity is a key factor in the pathogenesis of RSA. CYBB and LYN are regarded as the crucial genes that constitute a model and contribute to inflammation-immunity throughout the occurrence and progression of RSA. These findings provide a new perspective on the diagnosis and pathogenesis of RSA.

## Introduction

Recurrent spontaneous abortion (RSA) shows up prevalently and challengingly within the scope of obstetrics and gynecology (1). The Royal College of Obstetricians and Gynecologists (RCOG) sets the definition of RSA as the happening of three or more natural miscarriages prior to the 24th week of pregnancy. However, the American Society for Reproductive Medicine’s criteria states that it is two or more natural miscarriages (2). In China at present, RSA is defined as the occurrence of three or more natural miscarriages within 28 weeks of pregnancy with the same partner (3). Not only does this condition augment psychological pressure and diminish self-esteem, resulting in psychological issues such as anxiety and depression, but also frequent abortions tend to induce endometrial abnormalities. It is shown by epidemiological survey data that a history of RSA can be found in around 1%–5% of women during their reproductive years (4). Considerable proof points to the fact that RSA is correlated with immunologic factor abnormalities, genetic defects, abnormal genital structure, endocrine disorders, inflammation, and other elements (5–7). Considering the existing absence of RSA screening, it is of great significance to identify innovative diagnostic biomarkers, which can help in the earlier detection of potential RSA, as well as to locate therapeutic targets to better the prognoses for RSA.

With the ongoing advancement of reproductive immunology in recent times, an increasing quantity of research evidence points to the fact that the occurrence of RSA is closely related to the imbalance within the maternal-fetal immune tolerance mechanisms. Two types of immune cells exist: the innate immune cells [neutrophils (8), Natural killer (NK) cells (9), myeloid-derived suppressor cells (MDSCs) (10) and macrophages (11)] and the adaptive immune cells (B (12) and T cells (13)). The disproportion of these immune cells may pose a threat to the success of pregnancy. The dysregulation of the immune system is likely to trigger immune cells to launch attacks on the embryo or the placenta (14).

Once human pregnancy initiates, the blastocyst which has hatched from the zona pellucida adheres to and is implanted within the endometrium of the maternal uterus. A sequence of crucial events, namely embryo implantation, decidualization, placentation, and parturition, is essential for the success of human pregnancy. In pregnancy, the physiological events are inflammatory processes. What is needed for the remodeling of intrauterine tissue, the feto-placental growth, and the parturition during the whole gestation is a balance of pro- and anti-inflammatory factors (15). NK cells play a vital role in initiating and resolving inflammation (16). Also, their existence is detected in each and every phase of pregnancy (17, 18).

This study aimed to uncover new DIIRGs related to RSA samples with the intention of pinpointing diagnostic Inflammation and immunity-related biomarkers by employing bioinformatics methods. Subsequently, we took further steps to confirm the DIIRGs within endometrium samples from both the normal pregnancy and RSA groups. Moreover, we explored the possible connection between the newly discovered DIIRGs and immune cells to promote further investigations into the origin and development of RSA in this particular area.

## Materials and methods

### Collecting and processing microarray data

We retrieved the relevant original PE datasets, namely GSE26787 and GSE165004, from the GEO database (https://www.ncbi.nlm.nih.gov/gds). It consisted of 30 Non-RSA and 34 RSA samples, as presented in **Table 1**. Based on the probe annotation files, in each dataset, probes have corresponded to gene symbols. When there are multiple probes corresponding to a single gene, the average expression value of those probes is taken as the expression level of that gene. The raw data underwent background correction and normalization by using the limma package of R (http://www.bioconductor.org/). Genes with |log fold change (FC)| = 0.585 and adjusted p < 0.05 were regarded as DEGs. Download the data of IRGs from the Import database (https://www.immport.org/shared/) (**Supplementary Table S1**), and download the genes related to inflammatory responses from the Molecular Signatures database (https://www.gsea-msigdb.org/gsea/msigdb) (**Supplementary Table S2**). Subsequently, the intersection operation was performed between the IRGs, inflammation-related genes, and DEGs, leading to the derivation of DIIRGs in RSA.

**Table 1:** mRNA expression profiles related to RSA from the GEO database.

Dataset ID	Platform	Non-RSA endometrium	RSA endometrium
GSE26787	GPL6244-17930	5	10
GSE165004	GPL16699-15607	25	24
Total		30	34

### Analysis of the function annotations and pathway enrichment for DIIRGs

The DIIRGs were subjected to GO and KEGG pathway enrichment analyses using the R packages “clusterProfiler”, “enrichplot”, “org.Hs.eg.db”, and “DOSE” with the aim of identifying the enriched GO terms within three distinct categories, namely cellular components, biological processes, and molecular functions, as well as the KEGG pathways. For visualizing the enrichment results, the “ggplot2” package in R was employed. A p-value less than 0.05 was regarded as the criterion for significant enrichment.

### Construction of protein−protein interaction

In order to obtain a PPI network, we made use of the STRING website (https://string-db.org/) (19), where 7 DIIRGs were entered into the “multiple proteins” module, and “Homo sapiens” was chosen for the organism module. The gene symbols were extracted from protein IDs and PPIs without corresponding gene names were filtered out. Subsequently, Cytoscape 3.10.0 was applied to build the PPI network. Additionally, the cytoHubba plugin assisted in spotting the hub genes.

### Constructing a diagnostic prediction model of RSA with DIIRGs

By means of Spearman correlation analysis to select DIIRGs possessing a |logFC| value of 0.585, the significant diagnostic biomarkers in RSA were pinpointed. The employment of the LASSO algorithm together with the mSVM-RFE algorithm facilitated the discovery of such biomarkers.

To conduct LASSO, which is an algorithm in regression analysis for variable selection to avoid overfitting, the “glmnet” package was applied. In addition, the mSVM-RFE algorithm was implemented using the “e1071” R package. This algorithm employs resampling methods in each iteration to stabilize feature rankings and identify the most pertinent features by removing the feature vectors generated by the SVM via supervised machine learning techniques (20). As mSVM-RFE has a lower risk of overfitting compared with SVM-RFE, we combined LASSO and mSVM-RFE to screen for overlapping genes, and then these genes were validated within the training set.

ROC curves were finally produced by means of the “pROC” R package. The ability of biomarkers to distinguish RSA from Non-RSA endometrium samples diagnostically was evaluated via the AUC of the ROC curve.

A nomogram model for the diagnostic capability of RSA was built and verified.

For the purpose of predicting RSA occurrence, the “rms” and “rmda” packages were employed to build a nomogram model. Wherein, each factor had its score marked as a “point,” and the sum of all factor scores was defined as “total points.” Then, calibration curves were produced to examine the nomogram model’s predictive capacity.

### Patients and tissue specimens

Evelen paraffin-embedded samples, which consisted of five Non-RSA endometrium and six RSA endometrium samples, were collected from the Second Affiliated Hospital of Fujian Medical University (Fujian, China). The approval for this study had been obtained from the Research Ethics Committee of the aforementioned hospital prior to its initiation.

### Immunohistochemistry

The IHC staining was carried out according to the procedures described previously (21). The primary antibodies, namely anti-LYN (18135-1-AP, Proteintech, USA), anti-CYBB (NOX2) (19013-1-AP, Proteintech, USA), and anti-cMET (MET) (AF6128, Affinity, England) were utilized. The staining intensity ratios of LYN, CYBB, and MET were classified into four levels: negative (scored as 0 points), light yellow (1 point), brownish yellow (2 points), and tan (3 points). Regarding the number of stained cells, it was evaluated based on the following proportions: if less than one-third, 1 point was assigned; if between one-third and two-thirds, 2 points; and if more than two-thirds, 3 points. The final expression scores of LYT, CYBB, and MET were calculated by multiplying the two aforementioned ratings. Subsequently, the slide samples were divided into two groups, namely the low-expression group and the high-expression group, which were defined by scores of less than 6 and greater than or equal to 6 respectively. The histopathological diagnoses of the patients were validated by two pathologists who specialized in obstetrics and gynecology.

### The distribution of immune cells in RSA and biomarkers

The quantification of the proportion of infiltrating immune cells from the gene expression profiles in RSA was achieved by employing the CIBERSORT algorithm (http://cibersORT.stanford.edu/). The LM22 gene signature matrix, downloaded from the CIBERSORT webpage, was utilized to estimate the putative abundance of immune cells (22). The “corrplot” R package was utilized for conducting the correlation and visualization of LM22. Pearson’s correlation analysis was adopted to explore the relationships of the screened diagnostic biomarkers with the levels of infiltrating immune cells, with the results being visualized by means of the “ggplot2” R package.

### Statistical analysis

Using R software (v.4.1.3), all the statistical analyses were performed. With the Mann–Whitney U test, comparisons between the Non-RSA and RSA groups were made. LASSO regression, the SVM-RFE algorithm, ROC analysis, Pearson’s correlation, and unpaired t-tests were involved in the analyses. Throughout these analyses, statistical significance was determined as p < 0.05.

## Results

### Study procedure

**Figure 1:**
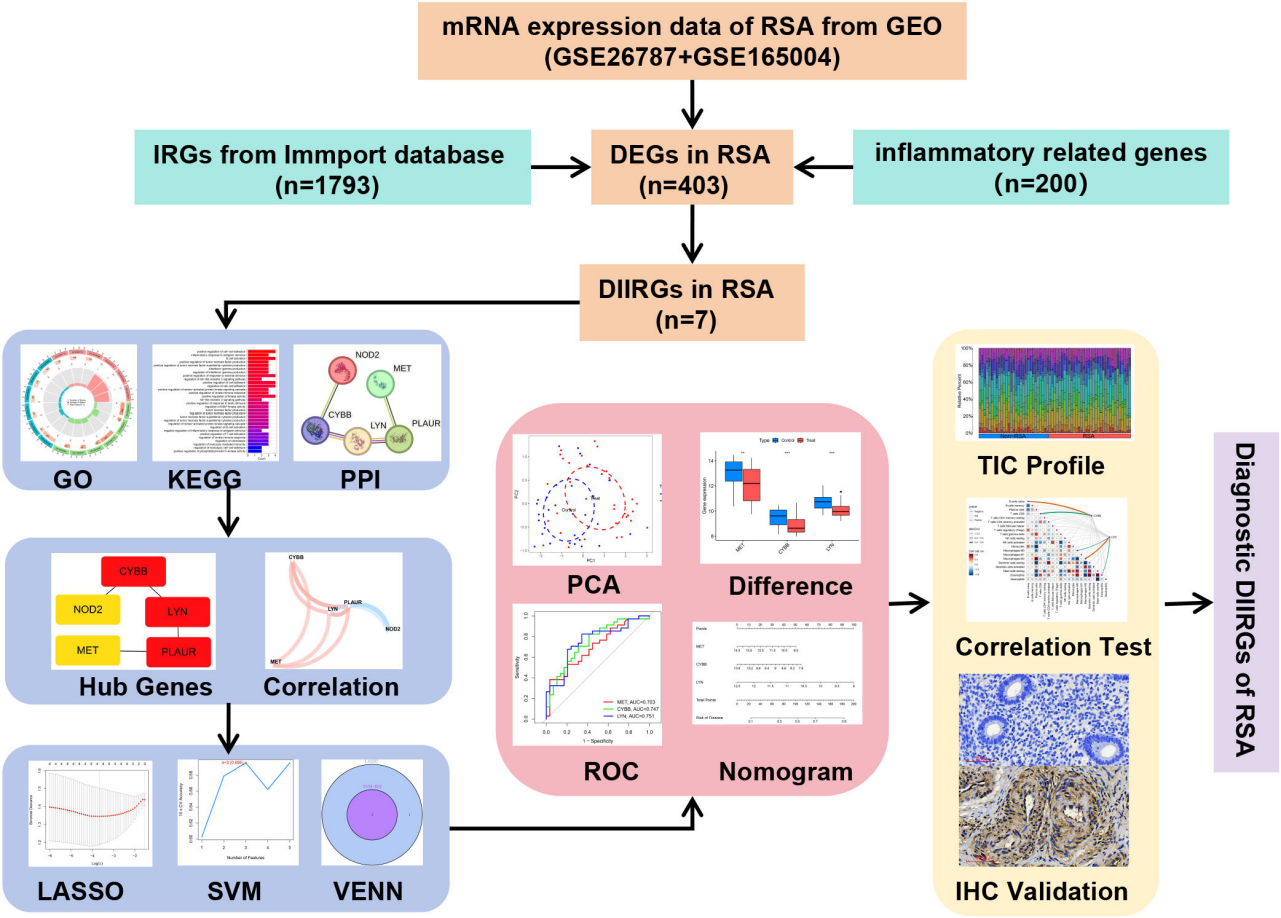
Process diagram of this research.

In the current study, the analytical procedure depicted in **Figure 1** was adopted. The transcriptome RNA-seq data were obtained from the GEO database. The screening of the overlapping DIIRGs between the Non-RSA and RSA groups was accomplished by utilizing conjugated DEGs inflammation-related genes and immune-related genes. Following this, GO, KEGG, PPI, and hub gene network analyses were performed on the identified DIIRGs. The overlapping DIIRGs were selected via the conjugated LASSO and SVM-RFE methods; ROC curves were generated to assess the predictive power of these biomarkers and were further validated with IHC. The compositional patterns of LM22 in PE were determined by the CIBERSORT algorithm. Additionally, a correlation analysis was conducted between the diagnostic immune-related biomarkers and the infiltrating immune cells.

### Identification of DIIRGs in RSA

**Figure 2:**
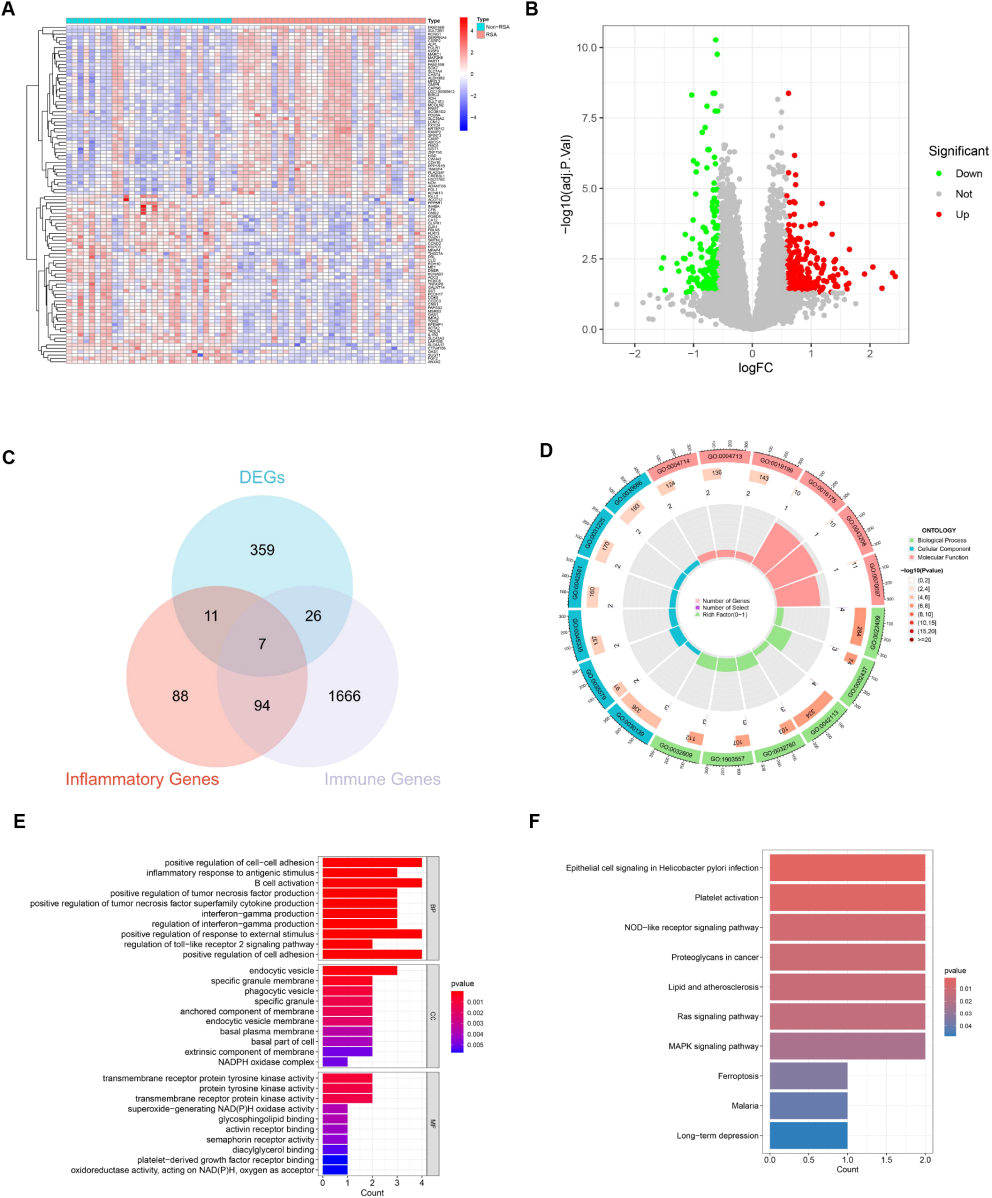
Identification and function of DIIRGs. **(A)** The expression levels of the first 50 DEGs between RSA tissue and Non-RSA samples from the GEO database are visualized through heatmaps. The genes are named in the row annotations, and the column annotations, which are the sample IDs, are not shown in the plots. The color gradient, which goes from red to blue, represents the expression levels from high to low in the heatmaps. **(B)** 403 DEGs between RSA and Non-RSA are illustrated by the volcano plots. In these plots, genes that are upregulated are indicated by red dots, genes that are downregulated are denoted by green dots, and genes without differential expression are represented by black dots. **(C)** 7 DIIRGs are contained in the Venn diagram of the intersection of differential genes, inflammatory genes, and immune genes. **(D)** A circle plot for GO analysis of 7 DIIRGs is presented. **(E)** A bar graph for GO analysis of 7 DIIRGs is shown. **(F)** The DIIRGs are annotated by KEGG.

With the utilization of the “limma” package in R, 403 DEGs between RSA patients and Non-RSA pregnancy within the GSE26787 and GSE165004 datasets were detected. The identification was based on the criteria of logFCfilter = 0.585 and adj.P.Val.Filter < 0.05 (**Figure 2A**). Out of these genes, 163 were notably downregulated and 240 were significantly upregulated (**Figure 2B**) (**Supplementary Table S3**). As depicted in **Figure 2C**, 7 DIIRGs were found through the intersection of the DEGs, IRGs, and the Inflammatory genes (**Supplementary Table S4**). Among the 7 DIIRGs, the expression of NOD2 was higher while the other 6 DIIRGs were remarkably downregulated in the RSA group.

### GO and KEGG functional enrichment

In order to gain a more in-depth understanding of the functions and enrichment pathways of the 7 DIIRGs, the “ClusterProfiler” package in R was employed to conduct functional enrichment analysis. The results showed that when it came to genetic biological processes (BPs), the focus was predominantly on positive regulation of cell-cell adhesion and inflammatory response to antigenic stimulus. The cellular components (CCs) of these genes were mainly located in the endocytic vesicle, specific granule membrane, and phagocytic vesicle. Regarding the molecular functions (MFs), they were primarily associated with transmembrane receptor protein tyrosine kinase activity, activin receptor binding, and superoxide-generating NADPH oxidase activity, with a significance level of P < 0.05 (**Figures 2D, E**, **Supplementary S1**). Additionally, KEGG enrichment analysis suggested that the 7 DIIRGs were mainly engaged in Epithelial cell signaling in Helicobacter pylori infection, NOD-like receptor signaling pathway, and Ras signaling pathway (P <0.05, **Figure 2F**, **Supplementary S2**). All these findings point to a significant correlation between RSA and inflammation-immunity.

### Construction of the PPI and hub genes network

**Figure 3:**
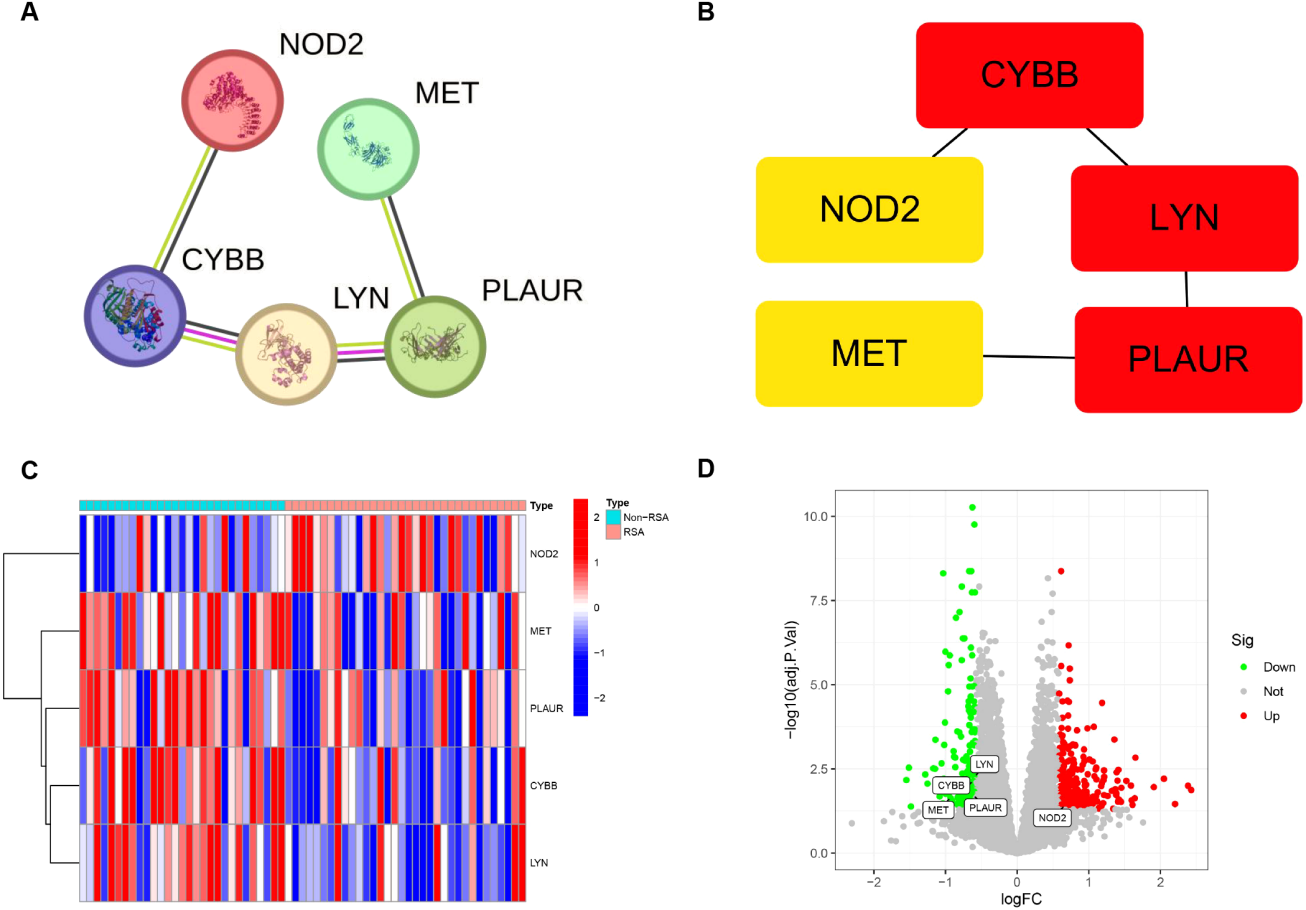
Association between DIIRGs and hub genes. **(A)** The PPI networks exploring five DIIRGs binding protein interactions are constructed by using the STRING tool. **(B)** Five hub genes are obtained through the MCC algorithm. **(C)** The expression levels of five hub genes between RSA tissue and Non-RSA samples are visualized through heatmaps. **(D)** Five hub genes between RSA tissue and Non-RSA samples are illustrated by the volcano plots.

The interactions of 7 DIIRGs were investigated by using the Search Tool for the Retrieval of Interacting Genes/Proteins (STRING) database (https://string-db.org/). Through this, PPI networks with concealed disconnected nodes were produced, and the remaining five genes (LYN, CYBB, PLAUR, MET, NOD2) have a connection (**Figure 3A**). After that, the cytoHubba plugin in Cytoscape software was utilized for clustering the genes in the network. Consequently, five nodes associated with the MCC were identified and clustered (**Figure 3B**). Notably, four of the genes (LYN, CYBB, PLAUR, MET) showed decreased expression levels, while NOD2 showed elevated expression in RSA group (**Figures 3C, D**).

### The linkages among the expressing magnitudes of five hubgenes in RSA

**Figure 4:**
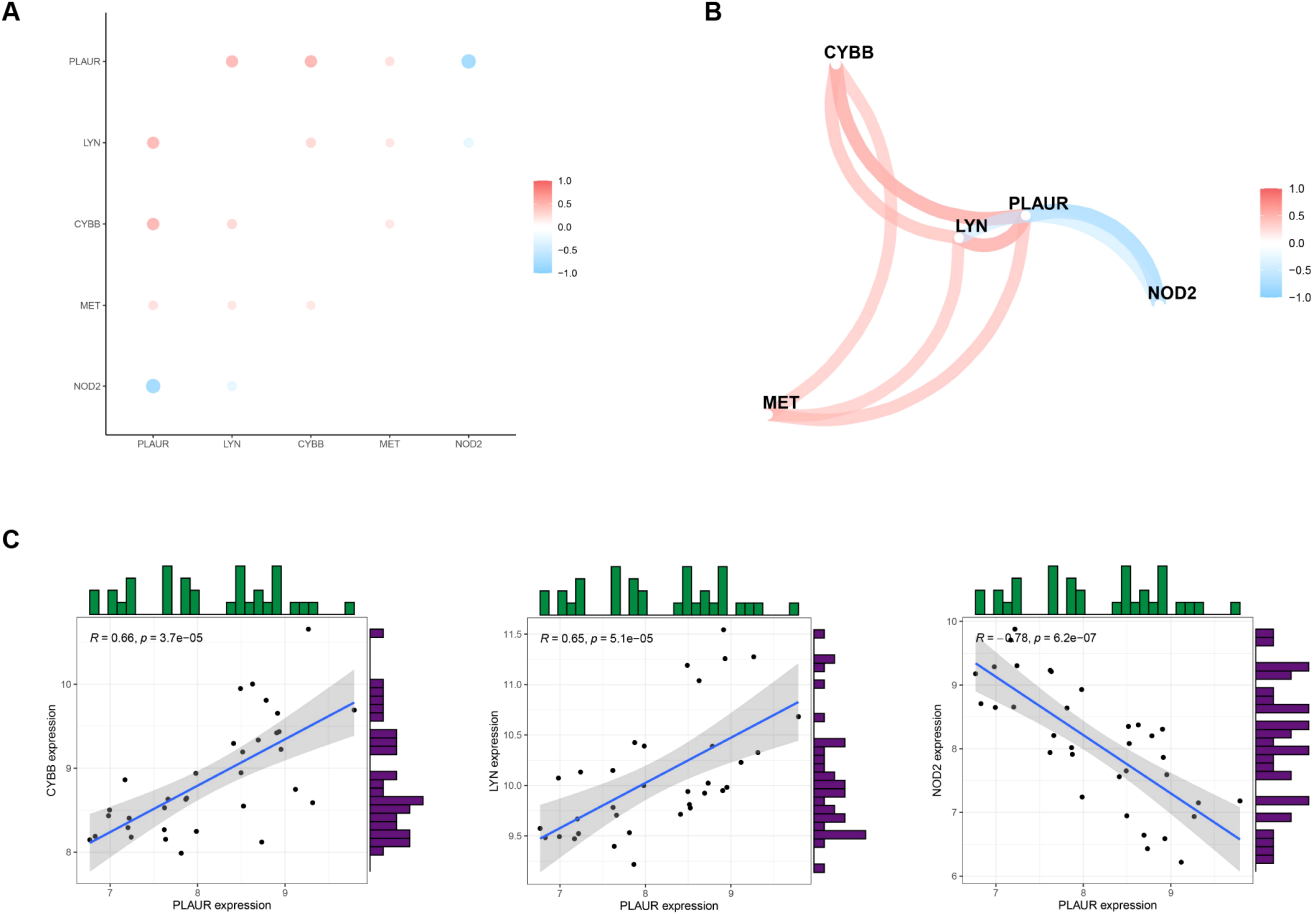
Analyzing the correlation of DIIRGs. **(A)** DIIRGs’ co-expression network map is depicted. **(B)** The correlation of DIIRGs is plotted. **(C)** A scatter plot for some highly correlated DIIRGs is provided.

To explore the correlations between the expression levels of the five DIIRGs, we carried out a correlation analysis with a cutoff of 0.5. By employing the “tidyverse” and “corrr” packages in R, we were able to visualize the results, thereby producing a coexpression network map (**Figure 4A**) and a correlation plot (**Figure 4B**). Moreover, scatter plots for the five gene groups with the highest correlations, where the cutoff was set at 0.6 and P<0.05 in RSA, were also generated (**Figure 4C**). It was found that PLAUR is positively correlated with LYN and CYBB (R_LYN_ = 0.66; R_CYBB_ = 0.65), while PLAUR shows a negative correlation with NOD2(R_NOD2_ = 0.66).

### A prediction model for RSA is under development

**Figure 5:**
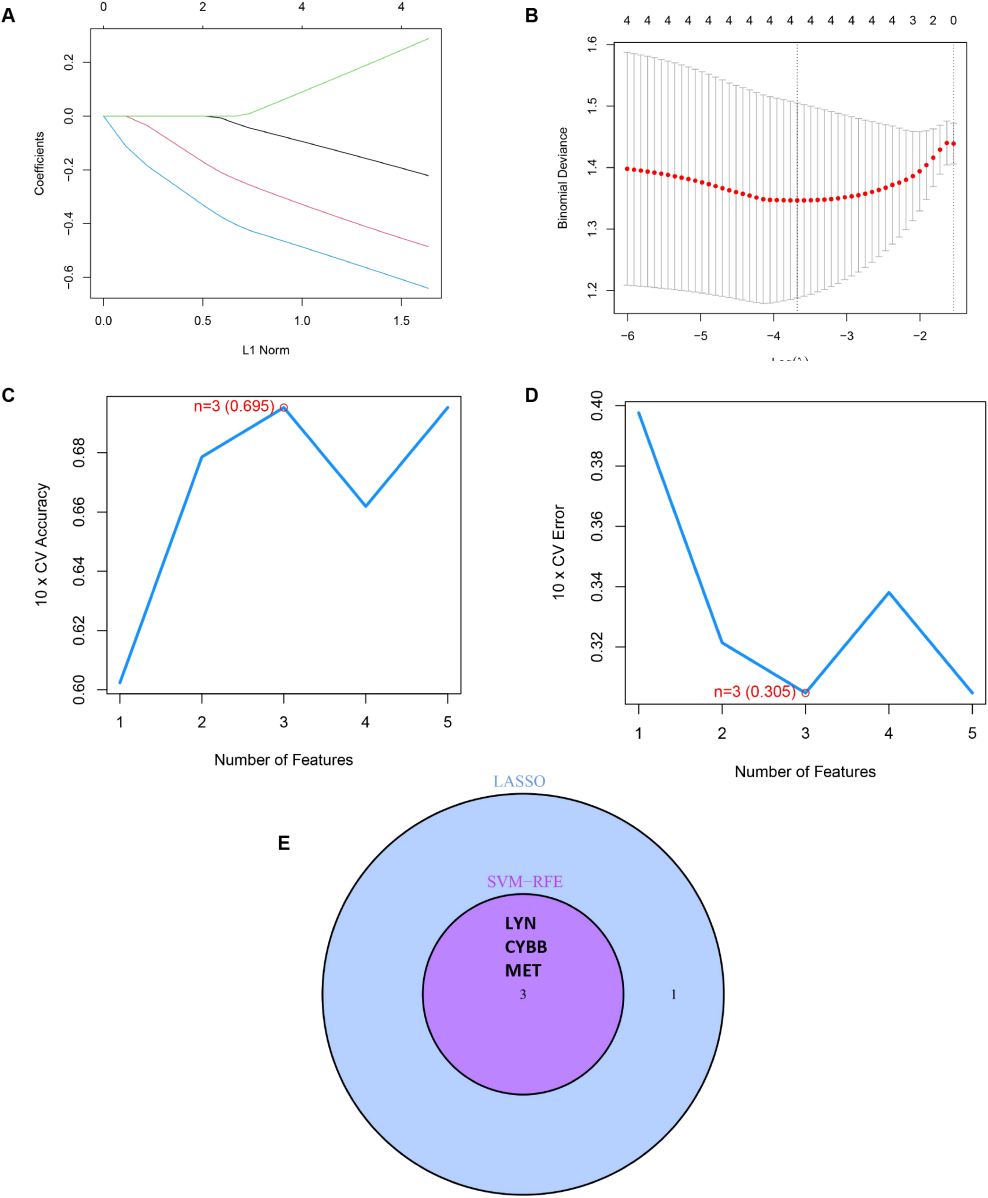
Construction of a prediction model for RSA. **(A)** A curve in the LASSO regression coefficient profiles of the 5 DIIRGs illustrates the changing course of each DIIRG. **(B)** The LASSO Cox regression model was employed to draw a plot of partial likelihood deviance versus log (l). **(C)** When k = 3, the curve of the total within the sum of the squared error curve under the corresponding cluster number k arrives at the “elbow point”. **(D)** At k = 3, the curve representing the average silhouette width for the corresponding cluster number k reaches its peak. **(E)** The Venn diagram shows the 3 diagnostic markers shared by the LASSO and SVM-RFE algorithms.

To accurately identify the key prognostic biomarkers in RSA, we utilized the LASSO and mSVM-RFE algorithms. Through LASSO analysis, four DIIRGs, namely MET, CYBB, NOD2, and LYN, were singled out from a group of five DIIRGs relevant to RSA, as shown in **Figures 5A, B**. Meanwhile, with the application of the mSVM-RFE model, this collection of five DIIRGs was further narrowed down to three DIIRGs, which included CYBB, LYN, and MET, as depicted in **Figures 5C, D**. When comparing the results of the two algorithms, it was found that the final selection consisted of three overlapping candidate genes: CYBB, LYN, and MET, as illustrated in **Figure 5E**.

### Further investigation into the three crucial DIIRGs

**Figure 6:**
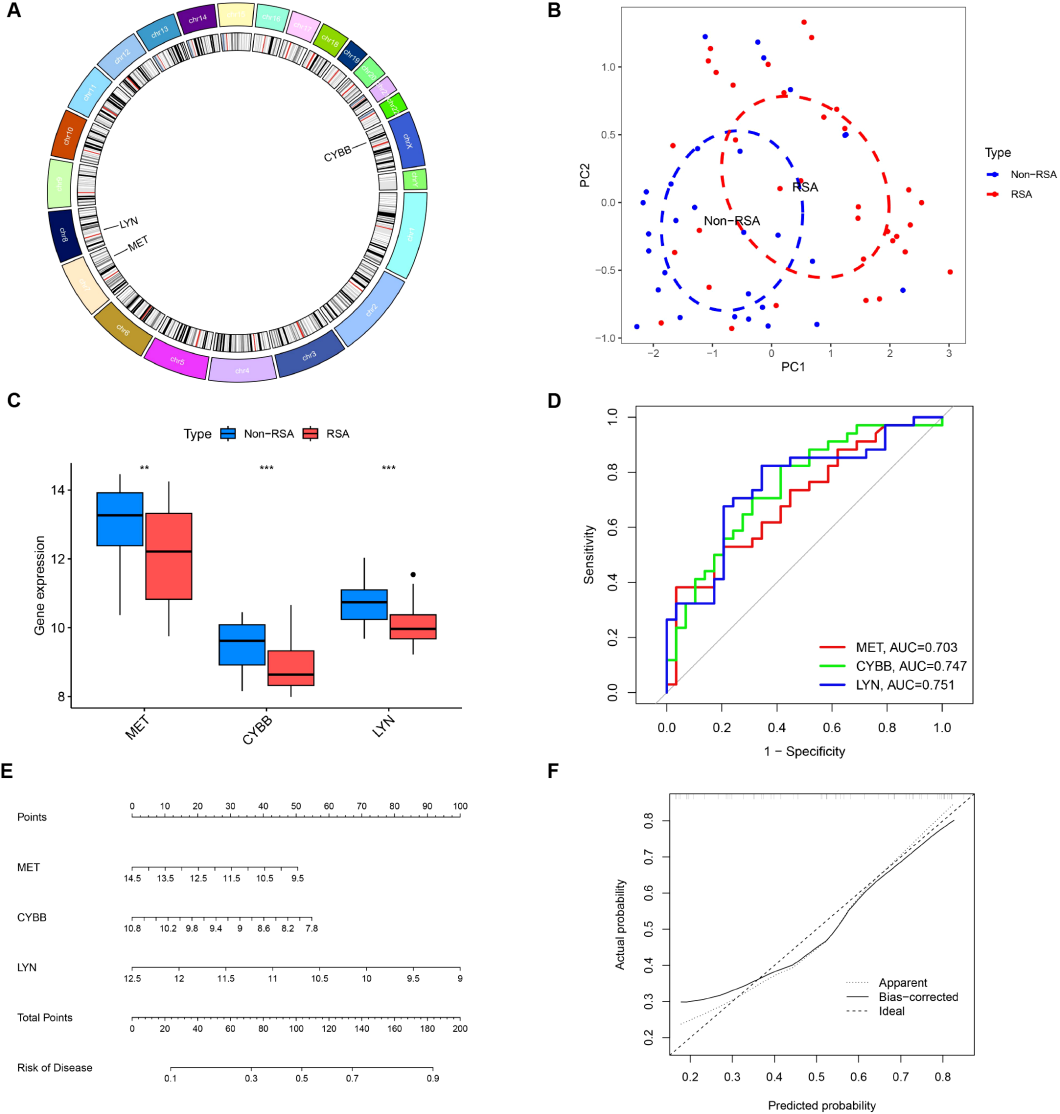
Additional analysis of three key DIIRGs. **(A)** The locations on the chromosome of three key DIIRGs. **(B)** RSA and Non-RSA can be clearly distinguished by principal component analysis using the three mentioned genes. **(C)** The relative expression levels of three key DIIRGs between RSA and Non-RSA are shown by the GSE26787+ GSE165004 datasets. **(D)** The performances of three key DIIRGs for predicting RSA in GSE26787+ GSE165004 datasets were verified by ROC curves. **(E)** Diagnostic Nomo plot related to three key DIIRGs. **(F)** Calibration curve of a model consisting of three key genes. **P < 0.01, ***P < 0.001.

Three candidate DIIRGs, namely CYBB, LYN, and MET, which emerged as the common elements found through both the LASSO regression model and the mSVM-RFE model, were picked out for further probing. In **Figure 6A**, the chromosomal locations of CYBB, LYN, and MET are presented. The findings from the principal component analysis imply that these three candidate genes are highly effective in discriminating between pregnancies with RSA and those of Non-RSA, hinting at a potentially crucial part they play in diagnosing RSA, as shown in **Figure 6B**. Additionally, the predictive effectiveness of these three genes was assessed, in contrast to Non-RSA pregnancies, CYBB, LYN, and MET exhibited downregulated expression in pregnancies affected by RSA (**Figure 6C**). The AUC values of the ROC curves for these three DIIRGs (AUC_CYBB_ = 0.747; AUC_LYN_ = 0.751; AUC_MET_ = 0.703) demonstrated a superior predictive power for RSA compared to Non-RSA pregnancies, as seen in **Figure 6D**. Subsequently, a risk-associated nomogram for RSA was devised (**Figures 6E, F**), which could function as a means to predict the capacity of the risk score to tell apart RSA from Non-RSA pregnancies.

### Verification of the three crucial DIIRGs

**Figure 7:**
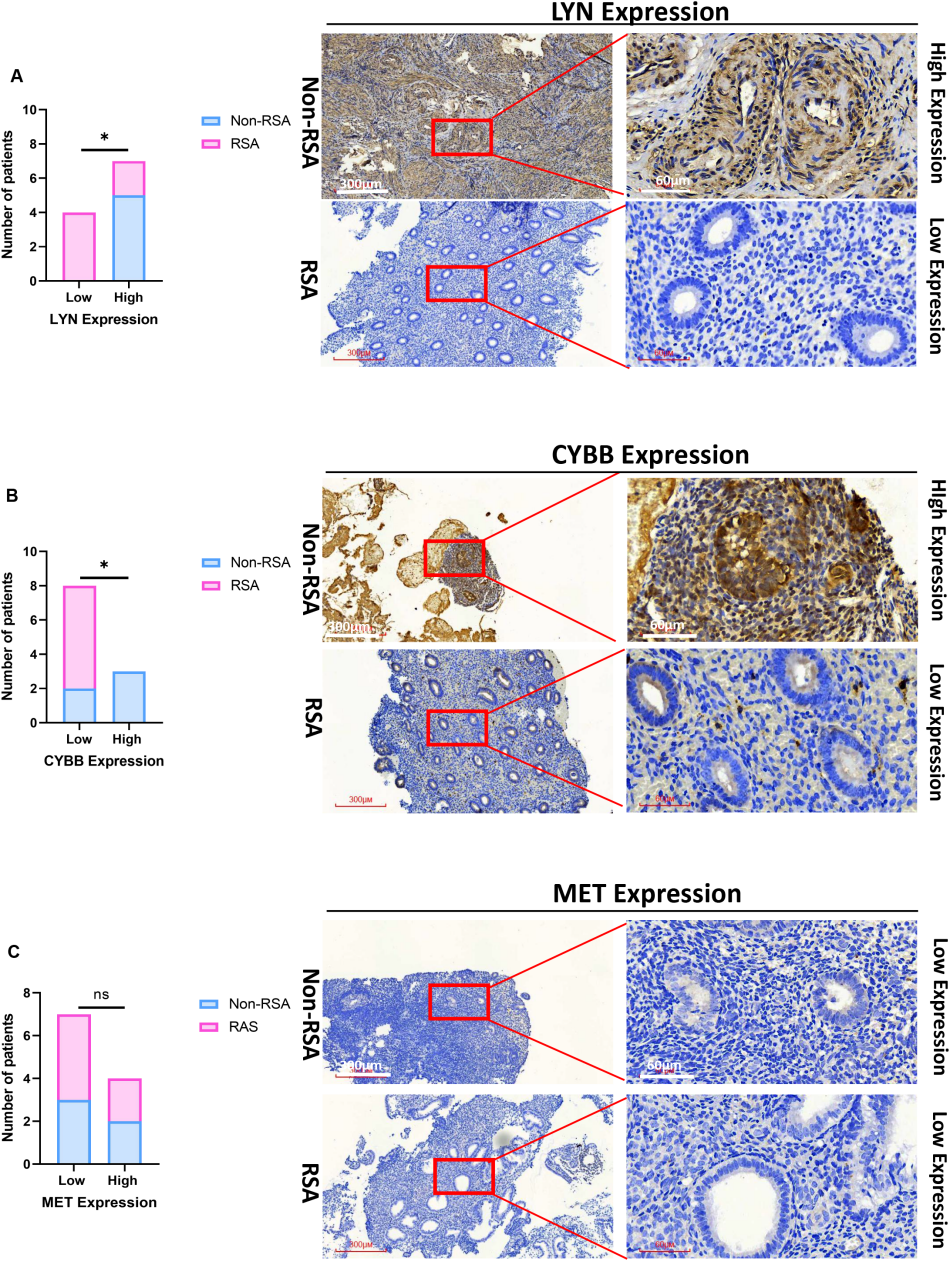
Validation of the three essential DIIRGs. **(A)** Significantly low expression of LYN was found in RSA tissues when compared with Non-RSA specimens (Non-RSA specimens = 5; RSA = 6). **(B)** Significantly low expression of CYBB was found in RSA. **(C)** No Significant expression of MET was found between RSA and Non-RSA tissues. Representative images (×40 and ×200) of IHC staining for LYN, CYBB, and MET in 6 RSA and 5 Non-RSA specimens (high expression versus low expression); *P < 0.05. ns, no significance.

For the sake of further clinical verification, we gauged the expression of CYBB, LYN, and MET by means of immunohistochemistry (IHC). The outcome disclosed that a low expression level of CYBB and LYN were correlated with RSA, both LYN and CYBB are expressed in the cytoplasm (**Figures 7A, B**; p < 0.05), the expression of MET was not significantly different between RSA tissues and Non-RSA tissues, it is also expressed in the cytoplasm (**Figure 7C**). Collectively, the aforementioned results imply that the CYBB and LYN genes exhibit stronger diagnostic potential.

### The distribution of immune cells is related to CYBB, LYN, and MET

**Figure 8:**
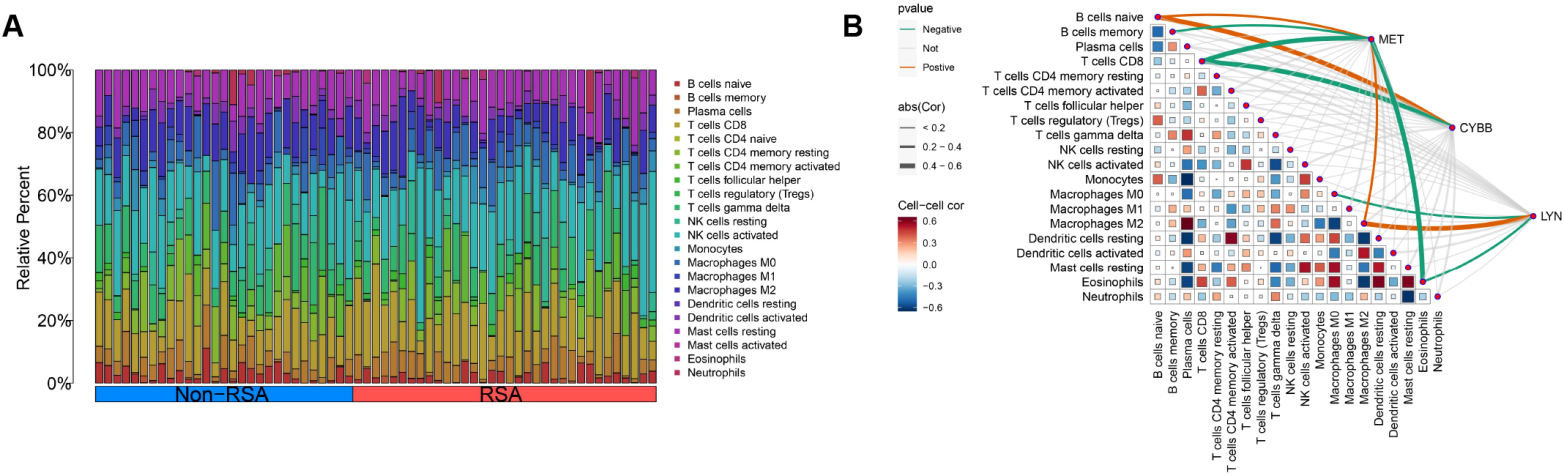
Distribution of immune cells is related to CYBB, LYN, and MET. **(A)** The distribution of 22 immune cell subtype proportions between RSA tissue and Non-RSA samples is illustrated by a bar plot. **(B)** Infiltrating immune cells in RSA are correlated with LYN, CYBB, and MET, and this correlation is analyzed.

In order to obtain a more profound understanding of the connection between immune cell infiltration and RSA, the CIBERSORT algorithm was employed to ascertain the relative proportions of 22 kinds of immune cells in both the control and RSA samples, as shown in **Figure 8A**. Subsequently, our exploration into the correlation between the CYBB, LYN, and MET genes and infiltrating immune cells yielded the following results. CYBB and MET were positively correlated with naïve B cells and negatively correlated with CD8 T cells, LYN and MET were positively correlated with M2 macrophages and negatively correlated with eosinophils, respectively (P < 0.05), as illustrated in **Figure 8B**. These findings in relation to immune activity bolster the influence of the CYBB, LYN, and MET genes.

## Discussion

RSA is a severe reproductive disease during pregnancy. It not only poses a great threat to the physical health of women of childbearing age but also brings heavy psychological burdens. Previous studies have shown that immunodeficiency accounts for the vast majority of the multiple factors leading to unexpected recurrent spontaneous abortions (23). Alloimmune RSA is usually referred to as unexplained RSA (URSA). Current research believes that it is related to abnormal immune cells, high histocompatibility of human lymphocyte antigens, and the lack of blocking antibodies (BA) (4). For a successful pregnancy, from the implantation of the embryo, the attachment of the placenta, the growth, and development of the fetus, to the delivery process of the fetus, a specific immune microenvironment is required, and the successful establishment of maternal-fetal immune tolerance is necessary.

GO enrichment analysis revealed that inflammatory response to antigenic stimulus and B cell activation in RSA. The results show that there is a close and non-negligible association between RSA and immune inflammation. Immune factors account for roughly 50% of the pathogenetic mechanisms in RSA (24). There exist a variety of immune cells including NK cells, macrophages, and DC cells at the maternal-fetal interface (25). In both maternal and fetal tissues, NK cells stand out as the immune cells with the highest abundance (26). Meanwhile, some immunotherapeutic approaches directed at NK cells have reaped promising achievements (27). The research suggested that the occurrence of RSA might be attributed to immune inflammation and oxidative stress brought about by metabolic dysregulation (28). Daher et al. (29) pointed out that for patients suffering from RSA, NK cell activity had increased and the levels of Th1-type cytokines including IFN-r and TNF-a had gone up. And they connected these observations to immune inflammation. Elevated levels of tumor necrosis factor-α (TNF-α), interleukin-6 (IL-6), and Th17/Treg ratios can be detected in pregnant women with URSA. Such elevations may give rise to an immune imbalance at the maternal-fetal interface and consequently increase the likelihood of miscarriages (30). This finding reveals that the occurrence of RSA is not caused by a single factor, but is rather a complex pathological process involving multiple cell types and a variety of internal and external factors working together.

KEGG enrichment analysis mainly involves NOD-like receptor signaling pathway and Ras signaling. Nucleotide-binding and oligomerization domain (NOD)-like receptors (NLRs) play a central part in both innate and adaptive immunity and they are intracellular proteins. Given that NLRs are members of pattern recognition receptors (PRRs), they can sense particular pathogen-associated molecular patterns. Subsequently, they initiate a multitude of signaling pathways, culminating in the secretion of diverse cytokines (31). It has been reported that the loss of NOD1 can heighten the inflammatory responses and modulate the pro-inflammatory reactions triggered by H. pylori infection (32). Jamontt et al. conducted a study and discovered that in IL-10-deficient mice, NOD2 signaling served to exacerbate colitis. Moreover, upon the deletion of NOD2 in IL-10/mice, a remarkable improvement in the symptoms of colitis was observed (33). It can be concluded from the above studies that this pathway is related to both inflammation and immunity. Currently, there is very little research on the NOD-like receptor (NLR) signaling pathway in RSA. NLRP7 is a member of the NLR family and a gene related to the NOD-like receptor (NLR) signaling pathway, serving as an immune sensor in innate immunity. In an *in vitro* decidualization model, the expression level of NLRP7 was elevated and it was translocated into the nuclei of endometrial stromal cells. When NLRP7 was overexpressed, the expression of IGFBP-1 was enhanced and the activation of the PR reporter was promoted (34). These findings indicate that NLRP7 plays a role in the *in vitro* decidualization process of endometrial stromal cells. The Ras signaling is one of the core pathways that regulate cell proliferation, differentiation, survival, and metabolism, and it is widely involved in biological processes such as development, immunity, and cancer. One study determines that alterations in the RAS pathway drive transcriptional remodeling of hematopoietic stem and progenitor cells (HSPCs) and the monocytic populations derived from them (35). Inflammatory signals, both intrinsic and extrinsic to the cells, act in concert with these mutations, ultimately resulting in the dysfunction of immune cells (35). Intracellular CD59 regulates the subcellular compartmentalization of Ras, which confers spatial selectivity to T - cell activation and presents a potential immunotherapeutic approach mediated by T cells (36). Up to now, there has been still limited research on the Ras signaling pathway in recurrent miscarriage. URSA is caused by abnormal H3K27 histone methylation of the RASA1 gene, which regulates the Ras-MAPK pathway in trophoblast cells (23). Cell cycle arrest and morphological changes in mouse embryonic fibroblasts deficient in RAS proteins are prevented by active R-RAS2/TC21 (37).

Based on the 7 DIIRGs that exhibited the most significant differences between the RSA and normal control groups, the LASSO regression and mSVM-RFE models were employed to screen out three genes, namely CYBB, LYN, and MET. Subsequent validation verified that the CYBB, LYN, and MET genes are correlated with RSA, as demonstrated by an area under the curve (AUC) greater than 0.7. CYBB, which can be found on the X chromosome, is precisely the gene that encodes the sizable gp91phox subunit of cytochrome b558. It should be emphasized that this cytochrome b558 constitutes the transmembrane moiety of the NADPH oxidase complex (38). It has been verified by research that chronic granulomatous disease (CGD) is the earliest discovered ailment related to CYBB. Specifically, CGD represents a primary immunodeficiency concerning phagocyte function, which stems from a deficiency in NADPH oxidase (phox) (39). Moreover, on account of mutations in CYBB, 70% of the cases show an X-linked pattern, consequently giving rise to a defect in the production of gp91PHOX (40). It has recently been demonstrated that CYBB/NOX2 within cDCs is instrumental in enhancing antigen presentation, thereby activating CD4+ T cells and leading to tissue damage mediated by TH cells (41). In a model concerning hyperhomocysteinemia-induced renal harm, the activation of NLRP3 inflammasome, which was instigated by NADPH oxidase-driven redox signaling, led to the recruitment of immune cell infiltration. Eventually, this cascade of events gave rise to glomerular injury and sclerosis (42). These results gain additional backing from our study.

LYN, which belongs to the src family of non-receptor protein tyrosine kinases, is mainly expressed in hematopoietic tissues. It is believed, similar to other members of the src family, that Lyn takes part in signal transduction from cell surface receptors without intrinsic tyrosine kinase activity (43). It is the signaling pathway of Lyn that has drawn extensive attention in the research on inflammation (44), immunity (45), allergy (46), and tumor (47). Given that Lyn participates simultaneously in both positive- and negative-regulatory pathways, a comprehensive and clear mechanistic map of its function remains mostly absent. This is because the effects it exerts are not only complex but also vary depending on cell type. In the sepsis-associated acute kidney injury (SA-AKI) model, the deletion of the LYN gene exacerbates tubular injury. Its protective mechanism involves the inhibition of STAT3 phosphorylation and cell apoptosis. The over-activation of STAT3 promotes the inflammatory response, while LYN alleviates inflammatory damage by negatively regulating the STAT3 pathway (48). In the Alzheimer’s disease (AD) model, LYN directly binds to TLR4 and regulates the inflammatory and phagocytic functions of microglia. LYN deficiency enhances the TLR4-induced neuroinflammatory response and weakens the phagocytic ability of Aβ, leading to aggravated neuronal damage (49). In systemic autoinflammation (such as LE and Ptpn6me - v/me - v mouse models), LYN deficiency or functional abnormalities (such as the pTyr508His mutation) lead to the over-activation of the NF - κB pathway, triggering severe systemic inflammatory responses (such as fever, chronic urticaria, and hypercytokinemia) (50). In B cells, LYN maintains immune tolerance by regulating BCR signaling and CSK-mediated phosphorylation (51). Low LYN expression leads to the over-activation of B cells, promotes the release of pro-inflammatory factors (such as TNF-α), and exacerbates the inflammatory response (52). The low expression of the LYN gene usually leads to reduced inhibition of inflammatory signaling pathways (such as TAT3, NF-κB, and TLR4), thereby promoting the inflammatory response and tissue damage. Our research findings indicate that LYN is lowly expressed in RSA, the finding is similar to the research mentioned above. ICAM1 activation alone can trigger eructophagy in macrophages via Lyn kinase (53). However, another study has indicated that Lyn plays a minimal part in the macrophage response to TLR4 activation (54). It appears that Lyn plays a more prominent positive part in neutrophil migration and trafficking. At the site of infection, peroxides are produced and they oxidize the cysteines within Lyn. This oxidation process triggers Lyn activation, subsequently guiding neutrophil migration to follow the peroxide gradient and move toward the infection site (55). To sum up, the LYN gene plays a dual role (either pro-inflammatory or anti-inflammatory) in the inflammatory response, the immune response of macrophages, and disease progression by regulating pathways such as TAT3, TLR4, and metabolic reprogramming. The specific effects depend on the microenvironmental signals and the types of diseases. There has been no report on LYN in RSA so far, and the mechanism of the role of LYN in RSA still needs to be further investigated.

CYBB encodes the gp91 phox subunit of the phagocyte NADPH oxidase, which is highly expressed in all phagocytes and lowly expressed in B cells (56). The CYBB gene is located on the X chromosome, and its mutation rate accounts for approximately 60-80% (57). The low expression of the CYBB gene is closely related to the occurrence and development of inflammation, and its mechanism of action involves the abnormal functions of various immune cells and the dysregulation of inflammatory signaling pathways. In the model of bronchopulmonary dysplasia (BPD), the expression of CYBB in alveolar M1 macrophages decreases, leading to the impairment of their pro-inflammatory functions. At the same time, it promotes the abnormal infiltration of macrophages in lung tissues, exacerbating inflammatory lung injury (58). In the microenvironment of pancreatic cancer, tumor cells induce the downregulation of CYBB in macrophages (such as U937 cells), promoting the polarization of macrophages towards the M2 phenotype (an anti-inflammatory/tumor-promoting phenotype). This state of low CYBB expression is associated with enhanced tumor-related inflammation and poor prognosis in patients (59). In the model of bladder infection, mice with NOX2 (CYBB) deficiency are unable to effectively inhibit the NF-κB signaling pathway due to insufficient production of reactive oxygen species (ROS). This leads to excessive infiltration of neutrophils and the loss of control of bladder inflammation (60). In colitis, CYBB cooperates with TLR4 to regulate the activation of the NLRP3 inflammasome. Low expression of CYBB may disrupt the inflammatory balance and exacerbate the damage to the intestinal mucosa (61). The inhibition of CYBB in zebrafish embryos leads to lipid metabolism disorders and excessive activation of the TLR4/NF-κB pathway, triggering liver injury and immune-inflammatory responses (62). The low expression of the CYBB gene weakens the activity of NADPH oxidase, alters the polarization state of immune cells, and activates inflammatory pathways such as NF-κB/NLRP3. Ultimately, this leads to the loss of control of the inflammatory response and tissue damage. This mechanism plays an important role in various pathological processes, including infectious diseases, autoimmune diseases, and the tumor microenvironment. Our research findings indicate that CYBB is lowly expressed in RSA, the finding is similar to the research mentioned above. It suggests that restoring the expression of CYBB or targeting its regulatory network may become a new strategy for treating inflammation-related diseases.

The MET gene, which is located on the long arm of human chromosome 7, region 2, band 1 (7q21 - 31), has 21 exons. Its encoded protein product is the hepatocyte growth factor receptor (HGFR). MET, which is a heterodimeric transmembrane tyrosine kinase receptor, is encoded by the mesenchymal-epithelial transition factor (*MET*) proto-oncogene and is mainly expressed in epithelial and endothelial cells (63, 64). Aberrant MET activation, which has been found in a broad range of solid tumors, facilitates uncontrolled cell proliferation, migration, and survival, leading to highly aggressive tumors (65). What’s more, several studies have demonstrated that the HGF/MET axis is necessary for the development and severity of different inflammatory and immune-mediated diseases such as colitis (66), COVID-19 (67), multiple sclerosis (68), and rheumatoid arthritis (69). An upregulation of colonic MET+ neutrophils during DSS colitis was demonstrated. The severity of DSS-induced colitis was reduced by the genetic ablation of MET in neutrophils. Concomitantly, the number of TH17 cells was decreased, which could be due to a decreased production of IL-1β by MET-deficient neutrophils (66). Intriguingly, a dual function appears to be fulfilled by the HGF/MET pathway in macrophage operations. The maintenance of a pro-inflammatory state associated with disease severity can be directly facilitated by the HGF/MET pathway (70, 71). However, it was also reported that the transition of macrophages towards an anti-inflammatory state promoting muscle repair is enhanced by HGF/MET (72). In our study, there is no significant difference in the expression of MET between RSA and normal pregnancy. MET was positively correlated with macrophages. This may weaken the anti-inflammatory effect of macrophages. Although there was no statistical difference in the verification of MET by IHC, we can increase the sample size in the future. Further investigation of MET on macrophages is needed in RSA.

Our research also has some drawbacks. Firstly, the study focused only on endometrial tissue, excluding other relevant maternal-fetal interface tissues (decidua or placenta) that could provide a more comprehensive understanding of RSA. After that, limited clinical heterogeneity (age, hormonal status and autoimmune background) were not incorporated into the analysis, which could affect the expression of immune and inflammatory genes. Due to the limited sample size, the nomogram model might need further verification prior to its clinical application. Subsequently,

limitations regarding one algorithmic approach for immune cell inference. Lastly, although the expression levels of CYBB, LYN, and MET have been confirmed by IHC, flow cytometry is still required to delve deeper into the action mechanisms of these molecules.

## Conclusion

To sum up, through the application of machine-learning methods, we probed into the possible association between Inflammation-immunity and the development of RSA. Our study uncovered a remarkable relationship between them. A novel predictive and diagnostic model related to inflammation-immunity composed of CYBB and LYN genes was discovered to have low expression levels in RSA. Additionally, this model is correlated with both immune cells and inflammatory cells. These findings may expand our understanding of the inflammatory response and immune regulation in patients with RSA, providing new insights into the diagnosis and treatment of RSA.

## Data Availability

The datasets presented in this study can be found in online repositories. The names of the repository/repositories and accession number(s) can be found in the article/**Supplementary Material**.

## Supplementary material

Supplementary Table 1 The immune-related genes (IRGs) from the Import database.

Supplementary Table 2 The genes related to inflammatory responses from the Molecular Signatures database

Supplementary Table 3 403 differentially expressed genes (DEGs) between RSA patients and Non-RSA patients within the GSE26787 and GSE165004 datasets

Supplementary Table 4 7 DIIRGs were found by intersecting the DEGs, IRGs, and the Inflammatory genes.

Supplementary S1 The result of GO enrichment

Supplementary S2 The result of KEGG pathway enrichment
