# Supplementary material for: LYN and CYBB are pivotal immune and inflammatory genes as diagnostic biomarkers in recurrent spontaneous abortion
Source: Front Immunol. 2025 Jul 7;16:1568536. doi: 10.3389/fimmu.2025.1568536 (PMC12278907; doi:10.3389/fimmu.2025.1568536)
Supplement: Supplementary Table 1 — The immune-related genes (IRGs) from the Import database. [file DataSheet1.zip › supplementary/raw material link.docx]

https://www.jianguoyun.com/p/DXvjgQEQ0dOWChiV_ekFIAA
